# Identification and Functional Analysis of Dual Nuclear Localization Signals on Desmin

**DOI:** 10.1021/acsomega.5c07336

**Published:** 2026-01-23

**Authors:** Ecem KURAL MANGIT, Pervin DİNÇER

**Affiliations:** † Hacettepe University, Department of Medical Biology, Faculty of Medicine, Ankara 06100, Turkey; ‡ Hacettepe University, Laboratory Animals Research and Application Center, Ankara 06100, Turkey

## Abstract

Traditionally regarded as a cytoplasmic scaffolding protein, desmin has recently been found to have nuclear functions alongside other intermediate filaments. Although several studies have reported desmin’s nuclear localization under specific conditions, the mechanisms behind its transport into the nucleus and the functional outcomes remain unclear. Computational analyses identified two putative bipartite nuclear localization signals (NLSs) within desmin’s sequence, suggesting it undergoes active nuclear import via the classical importin-α/β pathway. Analysis of GFP-fused deletion constructs in synchronized human skeletal myoblasts showed that disruption of these NLSs leads to a modest decrease in nuclear desmin levels, suggesting that they may contribute to nuclear localization Treatment with ivermectin, an inhibitor of the importin α/β pathway, resulted in a statistically significant but moderate reduction in nuclear desmin, supporting the notion that desmin translocation may involve karyopherin-dependent mechanisms. However, alternative karyopherin-independent transport mechanisms cannot be excluded, as the amphiphilic desmin may also directly interact with nucleoporins of the nuclear pore complex (NPC). Desmin contains two functional NLSs in its N-terminal and rod domains, mediating nuclear import. This nuclear transport is likely complex and may be regulated during myogenesis or cellular stress. Although subcellular fractionation is challenging due to desmin’s biochemical properties and tight perinuclear association, the findings provide new insight that may be crucial in muscle physiology and pathologies.

## Graphical abstract

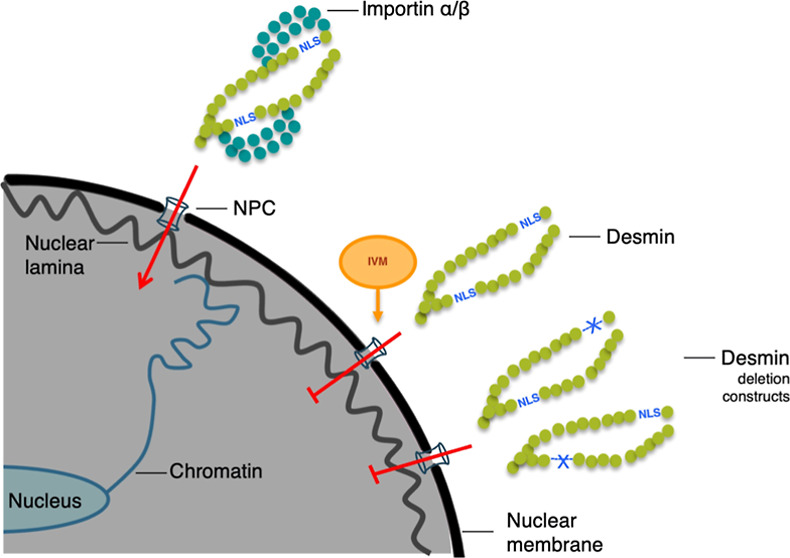

## Introduction

The compartmentalized organization of eukaryotic cells critically depends on nucleocytoplasmic transport, a highly regulated process that governs the spatial and temporal distribution of proteins and RNAs. This transport is mediated by the nuclear pore complex (NPC), a large multiprotein structure embedded in the nuclear envelope. While molecules smaller than 45 kDa can passively diffuse through the NPC,^1^ larger macromolecules require active transport mechanisms involving the karyopherin family of transport receptors.^1^ These receptors recognize nuclear localization signals (NLSs) and nuclear export signals (NESs) on cargo proteins, enabling direction-specific and signal-dependent transport between the nucleus and cytoplasm.^2^

In skeletal muscle cells, the proper localization of nuclear and cytoplasmic proteins is of particular importance due to the multinucleated nature of myofibers and their complex cytoskeletal architecture. Disruptions in nucleocytoplasmic protein distribution can impair transcriptional regulation, mechanotransduction, and cellular homeostasis. Notably, gene expression and protein localization can vary significantly between individual nuclei within the same myofiber, despite their shared cytoplasm; differential regulation of nucleocytoplasmic transport is a plausible underlying mechanism.^3^

Desmin is a muscle-specific type III intermediate filament (IF). It connects myofibrils to membranous organelles and facilitates force transmission during muscle contraction.^4,5^ As one of the earliest myogenic markers, desmin is detectable during the development of somites and cardiomyocytes.^6–9^ Mutations in the desmin gene (DES), which encodes desmin, cause skeletal and cardiac myopathies, collectively known as desminopathies.

Traditionally considered a cytoplasmic scaffolding protein, desmin has recently been implicated in nuclear functions, along with other intermediate filaments.^10–20^ Several studies have reported nuclear localization of desmin under specific conditions,^10–17^ such as in BHK21 cells where desmin was detected within the nuclear compartment,^10^ during cardiomyogenesis where amino-terminal deletion or serine-to-alanine substitution reduced Nkx2.5 expression and impaired cardiac differentiation,^17^ or in vitro, where desmin was shown to interact with G-rich single-stranded DNA and G-quadruplex structures.^12^ However, transport mechanisms and functional consequences of such nuclear targeting remain unclear.

In silico analyses have identified two putative bipartite nuclear localization signals (NLSs) within the desmin protein sequence,^21–23^ suggesting active import via the canonical karyopherin (importin-α/β) pathway.

To experimentally validate this, we employed robust immunofluorescence imaging and live-cell approaches to map nuclear presence of desmin with high resolution. Our data demonstrate that disruption of NLS significantly reduces nuclear accumulation of desmin, supporting their functional relevance.

Further, using ivermectin—a known inhibitor of the importin α/β pathway^24–26^ —we observed marked inhibition of desmin nuclear import, providing strong evidence that desmin nuclear translocation is karyopherin-dependent. While these observations underscore the active nature of desmin nuclear transport, we acknowledge technical limitations: efforts to biochemically fractionate nuclear and cytoplasmic compartments via Western blotting were hindered by desmin’s biochemical properties and its close physical association with the nuclear envelope. Accordingly, fractionation data were not considered in the interpretation of results, highlighting the critical reliance on imaging-based quantification for accurate analysis in this study. Our findings reveal a previously underappreciated nuclear dimension of desmin biology, highlighting potential nuclear roles during myogenesis or cellular stress. This work lays the foundation for future studies aimed at dissecting precise molecular interactions governing desmin transport, including its association with specific karyopherins or nucleoporins, and exploring functional outcomes of its nuclear localization in muscle health and disease.

## Results

### Nuclear Localization Signals in Desmin

Screening for potential NLSs in desmin was carried out using the cNLS Mapper database (https://nls-mapper.iab.keio.ac.jp/).^21–23^ According to cNLS Mapper, proteins exclusively localized in the nucleus have scores ranging from 8 to 10. For proteins mostly but not solely located in the nucleus, the score falls between 7 and 8; for proteins found both in the nucleus and cytoplasm, the score ranges from 3 to 5; while for proteins solely located in the cytoplasm, the score ranges from 1 to 2.^23^ Given desmin’s well-established cytoplasmic functions and the low a priori expectation of it harboring a classical NLS, we initially conducted an analysis using the “Entire region” setting with a cutoff score of 2. This revealed a putative NLS with a score of 3.2, encompassing glutamate at position 282 and alanine at position 313 (Figure 1).

**Figure 1:**
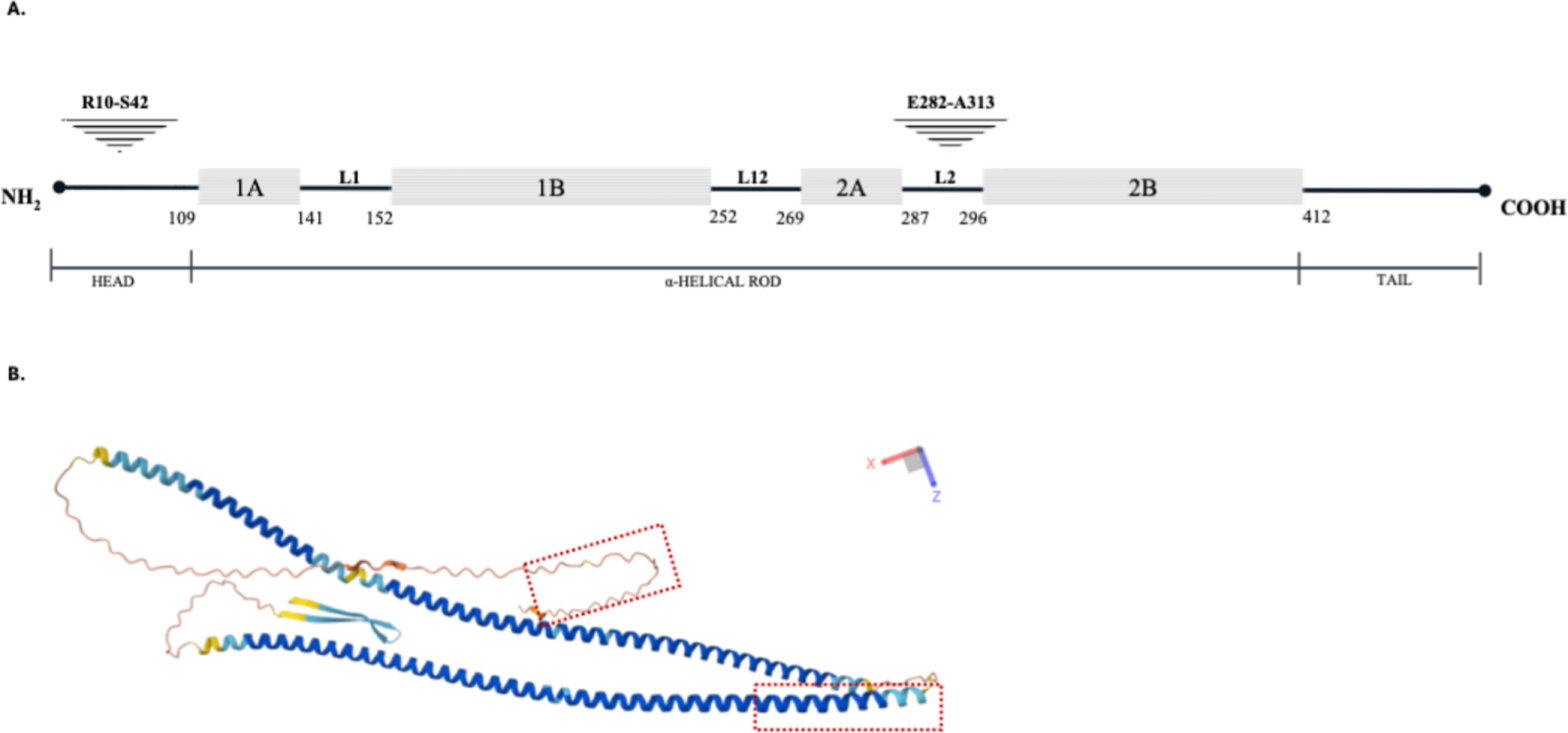
Putative nuclear localization signals (NLSs) in desmin. A. Schematic representation of three distinct domains: α-helical rod, the head, and the tail.^20^ The α-helical rod domain is segmented by three linkers (L1, L12, and L2), forming four coils (Coil 1A, 1B, 2A, and 2B). Both the head and rod domains are crucial for the intermediate filament (IF) assembly,^27,28^ whereas the tail domain appears to play a role in organizing the IF network.^27^ Triangles indicate the locations of NLSs (R: Arginine; S: Serine; E: Glutamic Acid; A: Alanine). Adapted with permission from A cytoplasmic escapee: desmin is going nuclear, Kural Mangıt E, Boustanabadimaralan Düz N, Dinçer P. Turkish Journal of Biology 2021; 45(6):711–719. Copyright 2021 TÜBİTAK. B. Approximative localization of the predicted NLSs on desmin (dotted red squares) on the model from AlphaFold Protein Structure Database (RRID: SCR_023662) (AF-P17661-F1).^29,30^ Structure predicted using AlphaFold^29,30^ and obtained from the AlphaFold Protein Structure Database (DeepMind and EMBL-EBI), CC-BY 4.0.

Furthermore, another potential NLS with a score of 2.9, located between arginine at position 10 and serine at position 42, was identified (Figure 1). Although the score of this second signal (score: 2.9) falls below the threshold defined by the software for proteins found both in the cytoplasm and the nucleus (threshold: 3), research by Fuchs (2016) demonstrated that the deletion of the first 48 amino acids at the amino terminus of desmin prevents its nuclear localization.^17^ This approach allowed us to identify potentially weaker but still functional NLSs, which may be contextually activated during muscle development or stress.

Thus, we considered both regions as candidate NLSs, consistent with both computational and experimental evidence.

### Cell Cycle Synchronization Enables Reliable Assessment of Desmin Nuclear Translocation without Affecting Myogenic Differentiation

Cell cycle synchronization is a critical step for the nucleocytoplasmic transport assays, as it ensures that the observed transport dynamics are not influenced by variances in cell cycle stages. Variations in the cell cycle can significantly impact nuclear envelope permeability, protein trafficking, and the availability of transport machinery, potentially introducing variability in the data. By synchronizing cells, we minimize this heterogeneity and create a more uniform physiological state across the cell population, allowing for a more precise assessment of nucleocytoplasmic transport processes.

Skeletal myogenesis is tightly coupled to the regulation of the cell cycle. Proliferating myoblasts exit the cell cycle to initiate differentiation, typically during the G1 phase. This commitment is associated with downregulation of cyclins and upregulation of muscle-specific transcription factors such as MyoD and Myogenin.^31^ Importantly, cells in S or G2/M phases are generally not competent for differentiation, as they are still engaged in the mitotic program. Therefore, synchronizing cells in G1 allows for enrichment of the population at a critical transition point, facilitating the study of molecular events associated with early myogenic differentiation.

To assess the nuclear translocation of desmin during the initiation of skeletal muscle differentiation, we synchronized cells specifically at the G1 phase. This approach enables us to capture early differentiation-specific processes, as the nuclear entry of regulatory proteins often coincides with the onset of transcriptional reprogramming in G1. G1 synchronization thus creates a defined and relevant cellular context to investigate mechanisms that are tightly coupled to cell cycle exit and myogenic commitment.

Although various methods exist for cell cycle synchronization, the approaches commonly employed to arrest the cell cycle typically involve serum deprivation. However, serum deprivation is not a preferred method as it triggers differentiation in skeletal myoblasts.^32^ Therefore, methionine, a nonessential amino acid vital for viability, was withdrawn from the culture medium. Cells were starved by maintaining them in the methionine-free medium during the period of doubling time. For the T0033 cell line, this period lasts 40 h.^33–35^ Following synchronization, cells were transferred into the standard growth medium for 24 h to allow them to re-enter the cell cycle. Subsequently, samples were analyzed using a flow cytometer. Amino acid starvation effectively synchronized the cells, resulting in their accumulation in the G0/G1 phase. Before treatment, only 15.9% of cells were in G0/G1, whereas after synchronization, this proportion increased markedly to 54.1% (Supplementary Figure 1). These findings demonstrate that the chosen starvation method was effective in cell cycle synchronization.

Fusion and differentiation indices served as morphological parameters to quantify myogenic differentiation. The differentiation index represents the ratio of MyHC-expressing cell nuclei to the total number of nuclei.^36^ The fusion index, on the other hand, is determined by dividing the number of nuclei in MyHC-expressing cells with at least two nuclei by the total number of nuclei.^36^

To show that the differentiation and fusion capacity of the cells were not affected by starvation, differentiation, and fusion indices were calculated at different time points. The differentiation rate was calculated as follows: 3% at 24 h, 7% at 28 h, 15% at 32 h, and 17% at 48 h after the induction of differentiation (Figure 2). Following induction of differentiation, the fusion rate was calculated as follows: 0% at 24 h, 6% at 28 h, 8% at 32 h, and 28% at 48 h (Figure 2). There is a consistent increase in both differentiation and fusion indices, indicating that the cells continued to proliferate and differentiate poststarvation as expected.

**Figure 2:**
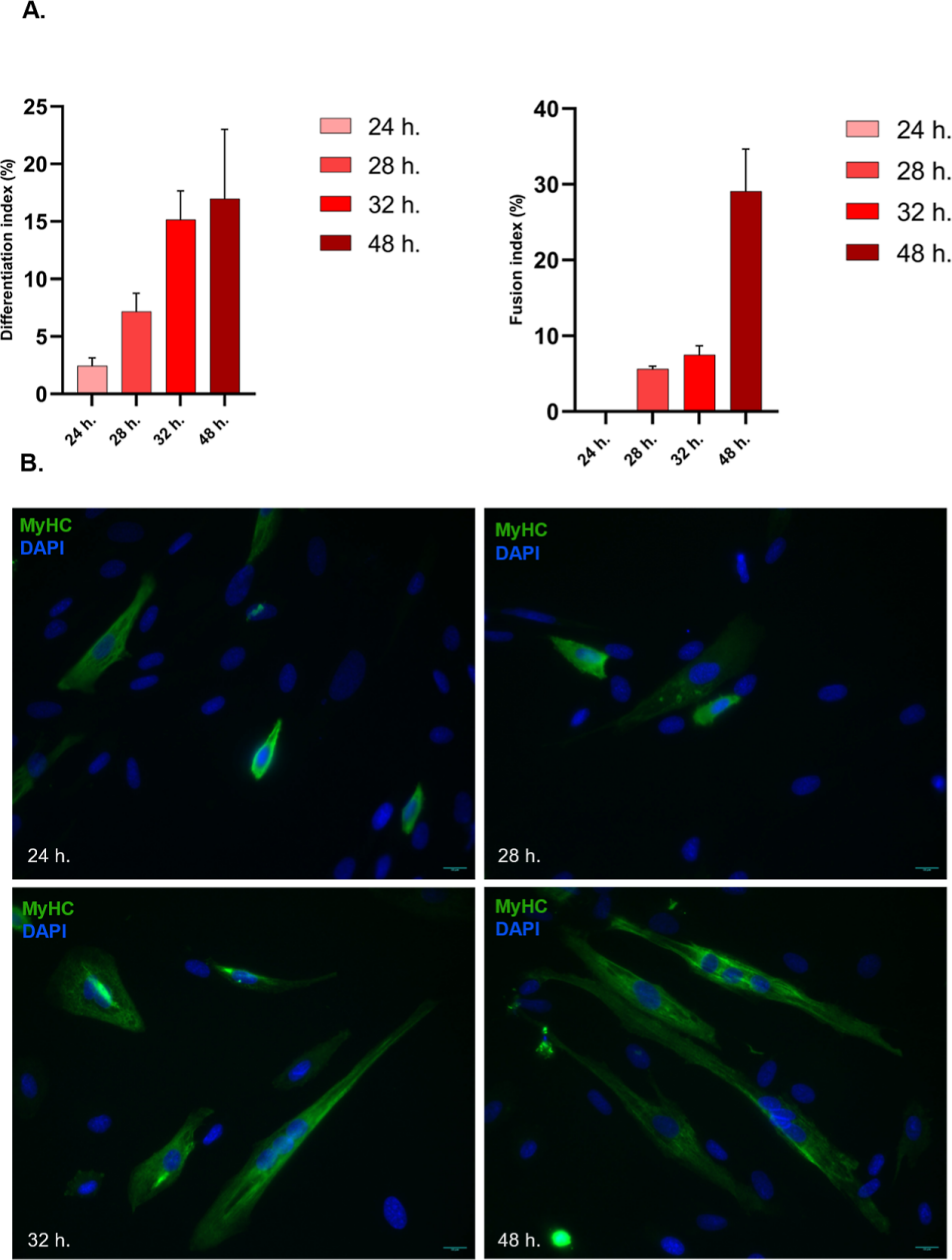
Skeletal myoblasts at the different time points of differentiation. (A). Histograms depicting differentiation and fusion indices. Following changing to differentiation medium, both fusion and differentiation indices exhibited a consistent increase. h: hours after induction of differentiation. Error bars were presented as mean ± SD. (B). MyHC staining at 24., 28., 32., and 48. Hours after the addition of differentiation medium. Scale bar: 10 μm.

### Desmin has Two Functional NLSs

To determine if the predicted NLSs on desmin were responsible for the nuclear translocation, cells were transfected with pCMV3-DES-GFPSpark and pCMV3-DES-GFPSpark ^Δ*R*10‑S42^ and pCMV3-DES-GFPSpark ^ΔE282‑A313^ (Figure 3).

**Figure 3:**
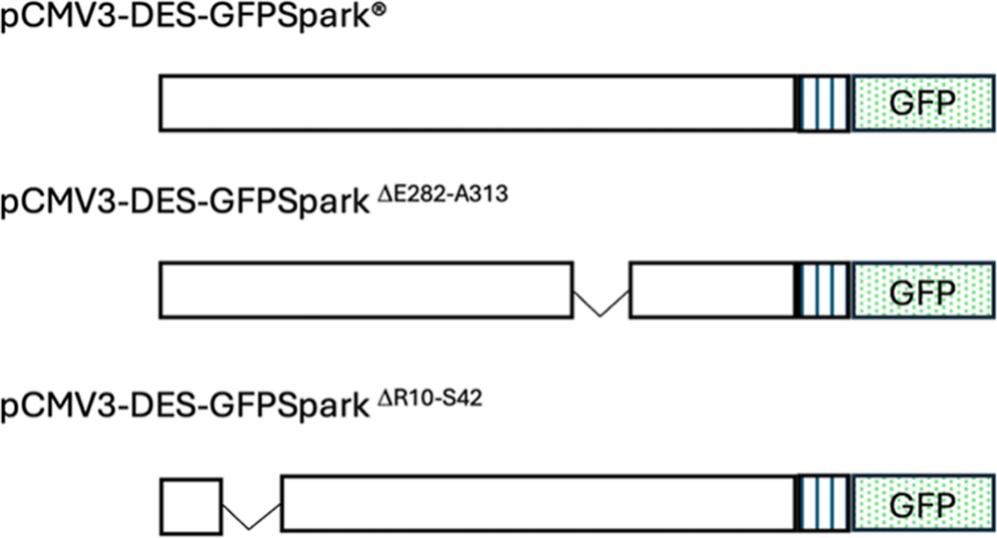
Schematic representation of the of the plasmids coding for full-length desmin (pCMV3-DES-GFPSpark) and NLS deletion mutants (pCMV3-DES-GFPSpark^Δ*R*10‑S42^ and pCMV3-DES-GFPSpark^Δ*E*282‑A313^) fused to GFP. Boxes with vertical lines indicate linker regions.

Based on an a priori power analysis assuming a medium effect size (Cohen’s *d* = 0.5) and 90% power, a minimum of 88 cells per group was required to analyze. Accordingly, a minimum of 101 cells per group were analyzed to ensure adequate statistical robustness.

In cells transfected with pCMV3-DES-GFPSpark, desmin was prominently observed within the DAPI boundaries (Figure 4). In contrast, cells transfected with pCMV3-DES-GFPSpark^Δ*R*10‑S42^ and pCMV3-DES-GFPSpark^Δ*E*282‑A313^, desmin was either not detected within the nucleus or exhibited very low signal intensity (Figure 4). We used the Pearson correlation coefficient (PCC) as colocalization indicator.^37^ PCC was significantly lower in cells transfected with pCMV3-DES-GFPSpark^Δ*R*10‑S42^ and pCMV3-DES-GFPSpark^Δ*E*282‑A313^ compared to the cells transfected with pCMV3-DES-GFPSpark (*p* < 0.0001) (Figure 4B). These results demonstrate that deletion of the putative NLSs disrupts desmin’s nuclear localization, confirming that these NLSs are functional and necessary for its nuclear import.

**Figure 4:**
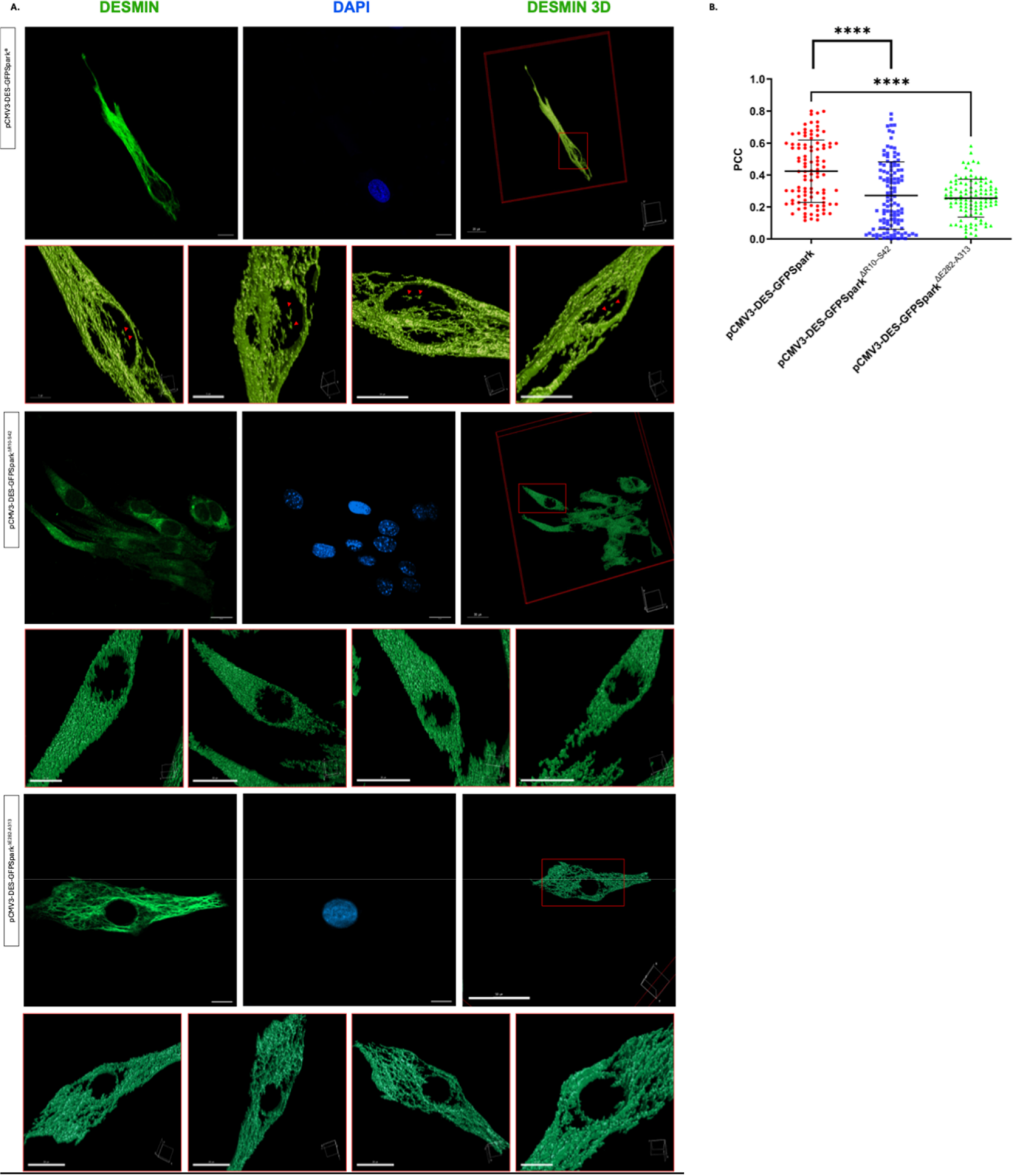
Localization of desmin in nuclei of human skeletal myoblasts. (A). Representative micrographs depicting the nuclear localization of desmin. In cells transfected with pCMV3-DES-GFPSpark desmin is prominently localized within the DAPI boundaries, while Δ*R*10-S42 and Δ*E*282-A313 desmin mutants (pCMV3-DES-GFPSpark^Δ*R*10‑S42^ and pCMV3-DES-GFPSpark^Δ*E*282‑A313^) show minimal or no localization. Third column shows 3D surface rendering of the same area. Confocal z-stack images were deconvolved using Huygens Professional, and surface rendering was performed with the Surface Renderer tool. In cells transfected with pCMV3-DES-GFPSpark, nuclear desmin fluorescence is observed independently of cytoplasmic extensions. In contrast, in cells transfected with plasmids encoding mutant sequences, desmin signal appears as a continuation of the cytoplasmic extensions without distinct nuclear localization. All images shown correspond to the same individual cell, presented using different projections or renderings. (B). Evaluation of colocalization in cells transfected with desmin mutants compared to full-length desmin sequence. Number of cells analyzed for pCMV3-DES-GFPSpark = 101; pCMV3-DES-GFPSpark^Δ*R*10‑S42^ = 118; pCMV3-DES-GFPSpark^Δ*R*10‑S42^ = 110. Mann–Whitney test was used to compare the PCC values (****: *p* < 0.0001). Error bars were presented as mean ± SD.

### Inhibition of the Nuclear Import Pathways Reduces Nuclear Localization of Desmin

Following the confirmation of the functionality of NLS sequences, cells transfected with pCMV3-DES-GFPSpark were treated with ivermectin, an agent targeting the importin α/β complex, to inhibit the nuclear import.^26^

After demonstrating that the deletion constructs exhibit reduced nuclear localization compared to full-length desmin, we performed an a priori power analysis to determine the number of cells required to detect the effect of ivermectin. Assuming a large effect size (Cohen’s *d* ≈ 0.8), a two-sided test with α = 0.05 and target power = 90%, the analysis indicated that approximately 33 cells per group are required.

Cells were transfected with pCMV3-DES-GFPSpark, followed by treatment with 25 μM ivermectin for 4 h post-transfection. Subsequently, cells were fixed and analyzed with confocal laser scanning microscopy, followed by colocalization analysis. PCC values indicate that the nuclear transport of desmin in cells treated with ivermectin was significantly lower compared to untreated cells (*p* = 0.0011) (Figure 5). These findings further support that desmin nuclear import relies on a karyopherin-dependent pathway mediated by its functional NLSs.

**Figure 5:**
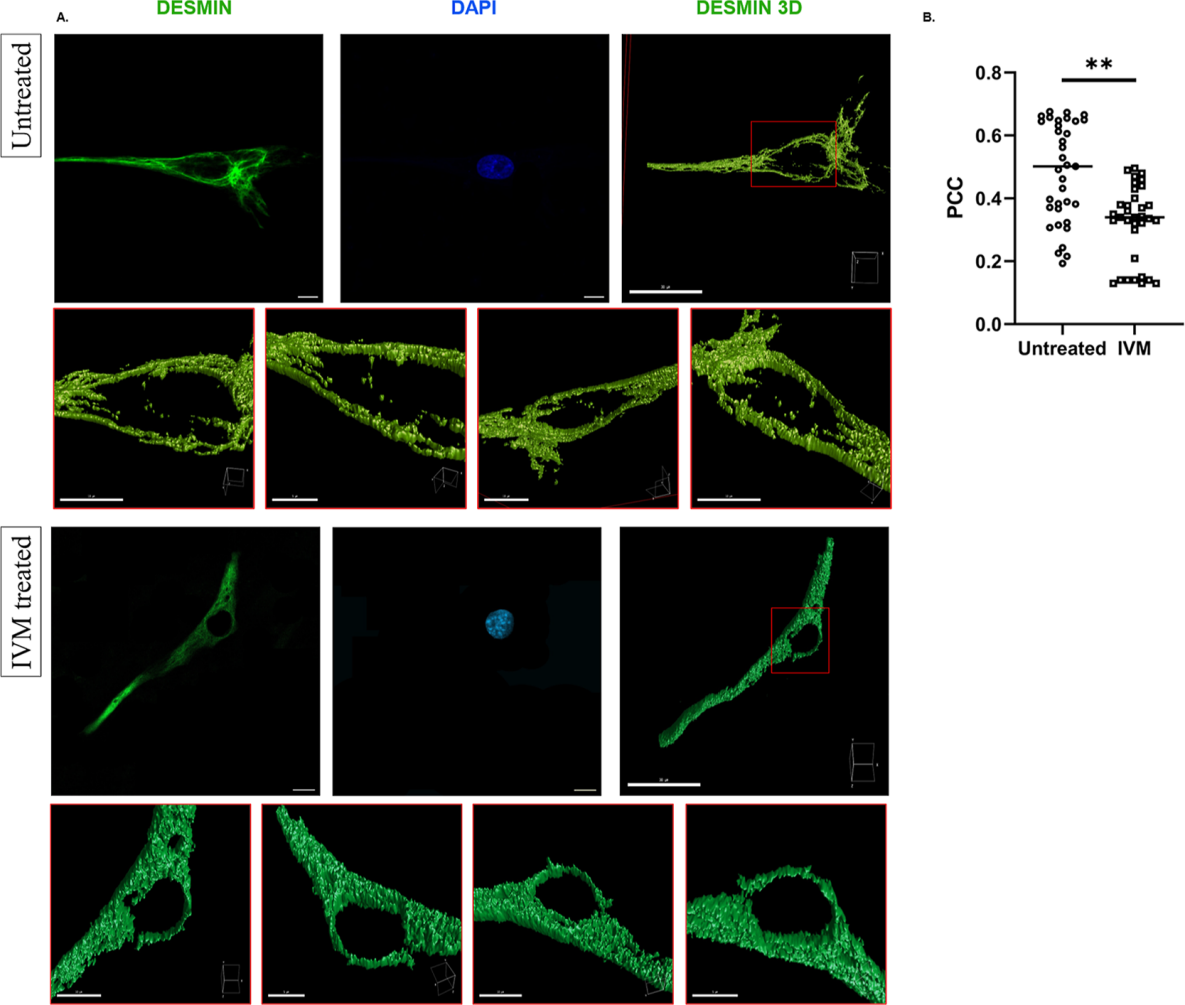
Effects of the ivermectin (IVM) treatment. (A). Representative micrographs and 3D reconstructions of human skeletal myoblasts with (IVM treated) and without (Untreated) IVM treatment. Scale bar: 10 μm. (B). Comparison of PCC values after IVM treatment in human skeletal myoblasts. The number of cells analyzed for each condition was 34. All images shown correspond to the same individual cell, presented using different projections or renderings. Student’s *t*-test was used for statistical significance comparison. (**: *p* < 0.01).

## Discussion

This study aimed to elucidate the nuclear transport mechanism of desmin, an intermediate filament (IF) protein traditionally associated with cytoplasmic structural roles. Our results indicate that desmin undergoes nuclear translocation through a karyopherin-dependent mechanism, mediated by at least two functional NLSs located in its N-terminal and rod domains.

Nucleocytoplasmic transport is intricately linked to the cell cycle and external signals.^38^ For instance, while the nuclear translocation of the transcription factor c-jun is not dependent on the cell cycle, the translocation of the v-jun is.^39^ Similarly, the nuclear translocation of lamin B2, an intermediate filament protein associated with the nuclear membrane during the interphase of the cell cycle, is linked to the phosphorylation of serine residues near the NLS.^40^ A previous study revealed significant sequence similarities between desmin and members of the helix–loop–helix (HLH) family, as well as with the basic and leucine zipper domains found in certain transcription factors.^41^ These similarities are associated with the possible involvement of desmin in signal transduction, transportation of specific myogenic factors to the nucleus, or the modulation of chromatin conformation.^41^ Although this study suggests potential functional similarities based on sequence resemblance to HLH or basic leucine zipper domains, the absence of structural conservation data warrants cautious interpretation of these assumptions. Another study proposed that desmin may activate myogenic HLH factors through association with lamin B.^14^ Fuchs et al. (2016) reported that desmin is involved in early cardiomyogenesis as part of a transcription factor complex.^17^ The evidence reviewed here suggests a pertinent role for desmin during early myogenesis.

The intracellular localization of proteins is a tightly regulated process essential for normal cellular function, particularly in muscle cells, where multinucleation and spatially restricted gene expression require precise control. Two prospective NLSs exist on desmin (Figure 1). To investigate the functionality of the signals, we deleted the NLS sequences on desmin and created mutant constructs. Each of the mutagenized constructs was transiently transfected into the human skeletal myoblasts. Nuclear accumulation of the mutant desmin constructs was significantly lower (Figure 4). These results indicate that both the NLSs are functional. Examples in the literature demonstrate the presence of multiple functional NLSs on a single protein.^42–46^ However, the present study was not specifically designed to determine whether these signals function independently or synergistically. To explore whether evolutionary conservation might inform the functional relevance of the two candidate NLS regions, we examined comparative sequence alignment across zebrafish (*Danio rerio*), chicken (*Gallus gallus*), mouse (*Mus musculus*), monkey (*Macaca fascicularis*) and human (*Homo sapiens*) (Supplementary Figure 3). While the predicted NLS spanning residues 282–313 is conserved across all analyzed species, the region encompassing residues 10–42 exhibits reduced conservation in zebrafish and partial divergence in chicken. Although sequence conservation alone is insufficient to infer functional necessity, this divergence is consistent with the possibility that the N-terminal region may contribute to nuclear targeting in a context- or lineage-dependent manner. Importantly, the presence of both conserved and less conserved targeting regions supports a modular nuclear import framework, in line with our experimental findings showing reduced -but not abolished- nuclear accumulation upon inhibition of classical import pathways. Although the presence of multiple functional NLSs on a protein has been reported in the literature, the reason for having multiple NLSs for a single protein remains unclear. Some studies suggest that different NLS sequences act together to enhance nuclear translocation efficiency,^47,48^ while others indicate that different NLS sequences may be recognized by different karyopherin family members in different cell types.^49^ The coexistence of multiple signals could also enhance nuclear translocation efficiency or enable context-dependent regulation. This may explain the observed overlap of the PCC values between constructs (Figure 4B), as distinct cellular states or requirements in multinucleated muscle cells might favor the activity of one or both NLSs. Although assessing importin α/β protein levels could further strengthen the mechanistic link, no evidence indicated altered expression of these transporters under our experimental conditions, suggesting that the observed effects are unlikely to arise from changes in their abundance.

Interestingly, although the introduction of a deletion of approximately 30 amino acids, a change that is expected to affect the protein function, this deletion did not appear to impact cell viability or proliferation. We also did not observe gross alterations in filament formation, although the effect on polymerization per se was not directly measured. Desmin’s ability to form filaments may be maintained through compensatory interactions with other cytoskeletal proteins. For instance, synemin can associate with desmin and stabilize the filament network even in the presence of mutations or deletions.^50^ Certain mutations in the non−α-helical carboxy-terminal domain of desmin, similar to those generated in our study, have been shown not to disrupt filament formation, indicating that some regions are more tolerant to structural alterations.^51^ In addition, other intermediate filament proteins, such as vimentin and nestin, can partially compensate for desmin loss or modification, thereby supporting overall network integrity.^52^ It should also be considered that the mutant desmin was introduced into cells that also express endogenous wild type desmin. In this context, the two forms of the protein are likely to coassemble, as desmin is known to form heteropolymeric filaments. This copolymerization could provide structural compensation, thereby preserving the overall filamentous architecture despite the mutation. Consequently, the impact of the mutant protein on filament organization and nuclear localization may be partially masked by endogenous wild type desmin. Experimental studies further demonstrate that some desmin mutants retain the capacity to assemble into normal filaments in vitro and in transfected cells despite substantial deletions or mutations, highlighting the robustness of desmin’s assembly mechanism.^53,54^

Pharmacological inhibition using ivermectin, a known blocker of the importin α/β pathway,^24^ led to a significant decrease in desmin nuclear signal, reinforcing the conclusion that desmin translocation is karyopherin-dependent (Figure 5). Nevertheless, our findings do not exclude the possibility of karyopherin-independent nuclear transport.

Multiple transport pathways for various proteins have been previously discussed in the literature. For instance, keratin, another intermediate filament protein, can be transported between the nucleus and cytoplasm via karyopherin-dependent mechanism,^55^ as well as through interactions with shuttling proteins.^55–58^ The possibility of keratin being transported between the nucleus and cytoplasm via shuttling proteins (piggybacking) is interpreted as enhancing the cell’s ability to adapt to the different physiological conditions and/or expanding the regulatory limits.^19^ Like all intermediate filament proteins, desmin possesses an amphiphilic region.^59^ Various studies in the literature have shown that amphiphilic proteins can be transported into the nucleus by directly interacting with the nucleoporins (nups), similar to members of the karyopherin family.^60,61^ When these proteins reach the nuclear pore complex, they expose their hydrophobic surfaces, interact directly with nups, and can be transported between the nucleus and cytoplasm.^60–63^ Previous research has established that desmin can interact with the FG-nups, namely nup153, nup214, and nup88.^64–66^ Various studies indicate that nup214, nup153, and nup88 are involved in the nuclear export of proteins.^61,67,68^ Furthermore, we also showed that desmin can undergo conformational changes in a hydrophobic environment.^69^ Desmin’s capacity to undergo conformational changes in response to hydrophobic environments supports its potential for direct interaction with the nuclear pore complex (NPC) via exposed hydrophobic patches, as has been demonstrated for other amphiphilic proteins.^60–63^ This dual transport potential may reflect a broader, adaptable nuclear transport mechanism employed by desmin depending on the physiological state of the muscle cell.

As in any tissue, the intracellular localization of proteins is crucial, as disruptions in localization impede the normal functioning of muscle. The maintenance of healthy muscle function is intricately linked to the spatial and temporal control of gene expression controlled by proteins shuttling between the nucleus and cytoplasm, such as transcription factors. Our results also intersect with the spatial dynamics of gene expression in multinucleated skeletal myoblasts. Previous studies, such as those by Cutler et al.,^3^ have shown that distinct nuclei within the same cytoplasmic environment can display different gene expression profiles, potentially regulated by differential nucleocytoplasmic transport mechanisms. The presence of multiple NLSs and transport routes in desmin may contribute to such spatial heterogeneity in muscle cells, potentially influencing development, regeneration, or stress responses.

Intermediate filaments are inherently polymeric proteins, best known for forming long, stable fibers in the cytoplasm. But what happens when they are inside the nucleus? do they retain their canonical filamentous architecture, or can they adopt alternative conformations more compatible with nuclear functions? Previous studies have shown that mutated forms of some keratins (K8/K18 and K8/K19) and vimentin can exist both as punctae and filaments, whereas K17 displays a punctate or diffuse nuclear pattern, demonstrating that IFs can occur in various nuclear forms. Hobbs (2016) further reported that the number and size of K17-containing nuclear punctae change depending on the cultured cells and suggested that it is likely that distinct cellular mechanisms act to suppress nuclear polymerization of endogenous keratins.^19^ In another study, nuclear and cytoplasmic K8 in HeLa cells had been shown to respond differently to conformation-specific antibodies and detergent extraction, indicating that nuclear keratins adopt a structure distinct from the filament structure in the cytoplasm.^70^ Similarly, the cytoskeletal proteins, actin and tubulin, exhibit unique nuclear conformations associated with specialized protein interactions.^71,72^ Based on these observations, Hobbs (2016)^19^ suggested a mechanism for keratin nuclear localization; nascent keratins in the cytoplasm first assemble into oligomeric precursors. These subunits can either integrate into mature filaments or undergo post-translational modifications that target them for nuclear import. Nuclear entry may occur through classical NLS–importin-α pathways or alternative noncanonical routes.^19^ These observations, together with reports proposing transcription factor–like roles for some IFs, make it conceivable that desmin might adopt similar functions. Nevertheless, clarifying the exact nuclear conformations of desmin and their potential functional relevance will require further studies, such as the use of conformation-specific antibodies as employed in prior studies.

Finally, considering the possibility that desmin may rely on a nuclear export mechanism, we also explored whether the protein harbors a putative NES. NetNES 1.1 analysis suggested a weak NES-like motif between two NLSs, which we experimentally tested by deleting this region (Supplementary Figure 2). Unexpectedly, the deletion did not increase nuclear accumulation but rather led to a decrease, arguing against a role as a classical NES. This indicates the motif is unlikely to function as a classical NES. Several explanations may account for this unexpected outcome. First, as highlighted earlier, NetNES and related tools rely on machine-learning algorithms trained predominantly on classical leucine-rich motifs.^25,73^ Because such patterns are common across the proteome, sequence-based predictions often produce false positives. Thus, the low-probability motif identified in desmin may represent such an artifact. Second, deletion of this region could have perturbed desmin’s tertiary structure, indirectly influencing its nuclear distribution. A misfolded or structurally altered protein may be selectively excluded from the nucleus if it cannot perform potential nuclear functions. Supporting this interpretation, analysis of mouse desmin (UniProt ID: P31001) in the same database did not reveal a candidate NES in the analogous region, even though the amino acid sequence between residues 192–200 is fully conserved. The discrepancy may arise from subtle nearby differences, such as residue 184 (isoleucine in mouse vs leucine in human) and residue 201 (arginine in mouse vs lysine in human), which could influence protein conformation and algorithmic recognition, as previously noted by La Cour et al. (2004).^74^

Another possibility is that the deleted region modulates the balance between nuclear import and export rather than acting as an export signal itself. Hydrophobic regions located near NLS motifs in some proteins are known to affect nuclear-cytoplasmic shuttling, and their removal can disrupt this equilibrium.^48^ A parallel has been described for Epstein–Barr virus DNase (EBV DNase), which contains two functional NLS motifs. Mutagenesis of adjacent hydrophobic regions selectively impaired one NLS while leaving the other intact.^48^ By analogy, the region in desmin predicted as an NES may instead regulate NLS activity, and its deletion may compromise nuclear import rather than export, explaining the observed reduction in nuclear accumulation. These observations further suggest that loss of residues 192–200 compromises nuclear targeting, raising the possibility that this segment functions as a nonclassical NLS. Although classical NLS motifs are typically recognized by the importin α/β complex, nonclassical signals can also associate with importin α, and importin β may mediate nuclear entry through alternative adaptor proteins. Thus, the importin pathway is not restiricted to canonical motifs. Furthermore, the affinity and specificity of importin α vary among isoforms and depend on the structural context of the cargo protein, emphasizing the versatility and complexity of nuclear import. In line with this framework, the observation that ivermectin reduces -but not abolish- nuclear accumulation of desmin is compatible with the possibility that multiple targeting mechanisms can influence its nuclear import.

In conclusion, although computational predictions suggested the presence of a weak NES-like signal in desmin, our data provide no evidence for its function as a nuclear export motif. The mechanism underlying the decreased nuclear accumulation remains unresolved. In summary, we demonstrate that desmin contains two functional NLSs and is imported into the nucleus through a karyopherin-dependent mechanism. Our findings highlight the potential complexity of nuclear transport in skeletal muscle cells and suggest that desmin may play nuclear roles during myogenesis or cellular stress. Further studies are needed to determine the regulatory mechanisms modulating desmin’s nuclear localization and its functional consequences in the nucleus.

## Conclusions

While this study provides compelling evidence for desmin’s nuclear transport and identifies two functional NLSs, several limitations remain. First, the study did not assess whether the two NLSs function independently or synergistically, which could be addressed using combinatorial mutagenesis and rescue assays. Second, although the involvement of the importin α/β pathway was demonstrated using ivermectin, specific karyopherin family members mediating desmin import remain to be identified. Third, potential cell-type–specific differences in desmin transport mechanisms were not explored, which may be critical given the specialized architecture of multinucleated muscle cells.

In addition, although Western blot-based compartmental analysis would have significantly strengthened our findings, desmin is a technically challenging protein to isolate. This limitation has also been noted in previous studies, where desmin’s biochemical properties hindered efficient fractionation.^75–82^ Despite multiple attempts using both commercial kits and manual methods, we consistently detected desmin in both fractions (For detailed information on methods and results, please refer to Supporting Information). This variability likely reflects technical limitations of the fractionation protocol in desmin-expressing cells, as desmin filament integrity interferes with clean nuclear extraction. While the results are not fully conclusive, they do provide biochemical evidence which is consistent with the nuclear localization observed by confocal imaging. Accordingly, representative blots are included in the Supporting Information to ensure transparency. Taken together, these observations support the presence of desmin in the nucleus, highlight potential noncanonical conformations, and underscore the technical challenges of biochemical validation, laying the groundwork for further mechanistic studies.

Future studies should aim to dissect the precise molecular interactions between desmin and specific importins or nucleoporins and explore whether different physiological or pathological states modulate these interactions. High-resolution live-cell imaging and proteomic analyses may further clarify the regulatory cues driving nuclear desmin transport. Comprehensive mechanistic and dynamic analyses, combining live-cell imaging, mutagenesis, and quantitative transport assays, will further clarify the mechanisms regulating nuclear desmin dynamics. Elucidating these pathways may not only refine our understanding of skeletal muscle biology but also shed light on the nuclear functions of cytoskeletal proteins in health and disease.

## Materials and Methods

### Cell Culture

Human skeletal myoblast cells (ABM Cat# T0033, RRID: CVCL_VG47) were cultured as described by Thorley (2016).^83^ Briefly, cells were grown in high-glucose DMEM supplemented with 16% Medium 199, 64% DMEM, 20% FBS, 25 μg/mL fetuin, 5 ng/mL hEGF, 0.5 ng/mL bFGF, 5 ng/mL insulin, 0.2 μg/mL dexamethasone, 50 μg/mL gentamicin, and 2.5 μg/mL amphotericin B (growth medium) at 37 °C in a 5% CO_2_ atmosphere.

### Cell Cycle Synchronization

To synchronize the cell cycle, 2 × 10^4^ cells were inoculated in a 6-well plate and supplemented with growth medium. After 24 h, cells were rinsed with 1X PBS and then shifted to starvation medium (methionine-free DMEM, 1% FBS, 1% l-glutamine, 40 mg/mL l-cystine).^84^ Cells were maintained in the starvation medium for 40 h. Subsequently, they were allowed to reenter the cell cycle by shifting to the growth medium.

Cells kept in standard growth medium for 24 h were subjected to cell cycle analysis to assess their synchronization status. Cells were harvested with trypsin. After the wash step with 1X PBS and vortexing, cells were fixed by adding 1 mL of cold absolute ethanol dropwise. The fixed cells were then incubated with the Muse Cell Cycle Kit for propidium iodide staining. Samples were analyzed with a CytoFlex flow cytometer device, ensuring at least 10000 events for each sample. Cell cycle analyses were performed with FlowJo v10 Software (BD Life Sciences, RRID: SCR_008520).

### Determination of the Fusion and Differentiation Indices

Cells were seeded on coverslips for immunofluorescence staining. After washing with 1XPBS, cells were fixed with absolute ethanol for 15 min at −20 °C. After two consecutive washings with 1X PBS for 5 min, cells were blocked and permeabilized in 1X PBS containing 1% FBS and 0.5% Triton X-100 for 1 h at room temperature. Cells were washed with 1X PBS, then incubated with the primary antibody against myosin heavy chain (MyHC) (DSHB Cat# MF 20, RRID: AB_2147781) for 2 h at room temperature. Cells were then washed 3 times for 10 min with 1XPBS. The secondary antibody, goat antimouse AlexaFluor 488 (Thermo Fisher Scientific Cat# A-11001 (also A11001, A 11001), RRID: AB_2534069), was diluted in 1X PBS and cells were incubated for 1 h at room temperature then washed 3 times for 10 min with 1X PBS. Cells were subsequently counter-stained with 4′,6-diamidino-2-phenylindole (DAPI) for 1 min. After washing with 1X PBS, cells were mounted.

Cells underwent differentiation in three separate experiments and were fixed at 24., 28., 38., and 48 h postdifferentiation. Following staining with MyHC and DAPI, ten fields per sample were captured using an Axio Plan upright fluorescence microscope (Carl Zeiss) equipped with an AxioCam Erc5 5Mp camera. Nuclei were manually counted on Fiji software (RRID: SCR_002285).

### Plasmids and Transfection

The plasmid encoding the full-length desmin protein (pCMV3-DES-GFPSpark, HG13865-ACG) was purchased from Sinobiological. Vectors encoding mutant desmin sequences (pCMV3-DES-GFPSpark ^Δ*R*10‑S42^ and pCMV3-DES-GFPSpark ^Δ*E*282‑A313^) were obtained from GenScript. Note that the abbreviation CMV in the vector names denotes the Cytomegalovirus promoter.

Synchronized human skeletal myoblasts were transfected when they reached 70–80% confluency. 200 ng of plasmid DNA was transfected using Lipofectamine 3000 (Thermo Fisher Scientific, L3000008) according to the manufacturer’s instructions. Twenty-4 h after transfection, cells were examined for GFP expression. Cells were fixed with 4% PFA, counter-stained with DAPI, and imaged using a Zeiss LSM 880 laser scanning confocal microscope equipped with a 63×/1.4 Apochromat Oil DIC objective.

Following transfection with pCMV3-DES-GFPSpark and a vector expressing only eGFP (pMAX-GFP, Lonza Bio), cells were subjected to trypan blue assay to assess the effects of transfection on cell viability as described earlier.^85^ Assays were performed in triplicate.

### Microscopy Image Processing and Analysis

Confocal microscopy images were processed using Huygens Professional software (version 25.04, Scientific Volume Imaging, The Netherlands). Deconvolution was performed using the Deconvolution Express tool, which applies an automated algorithm for point spread function (PSF) estimation and optimal signal-to-noise ratio (SNR) settings. Following deconvolution, chromatic aberration correction was applied using the software’s default alignment parameters, based on channel shift correction derived from the image metadata and instrument-specific calibration. All processing steps were performed using the software’s automated workflows to ensure reproducibility and standardization. The resulting images were used for downstream quantitative analyses, including colocalization measurements. The processed images were subsequently analyzed using Huygens Colocalization Analyzer Wizard. The Pearson correlation coefficient (PCC) was used as colocalization index.^37,86^ GraphPad Prism (RRID: SCR_002798) was used for statistical analysis and generating graphs. Data were considered statistically significant when *p* < 0.05.

### Inhibition of the Nuclear Import Pathway

Cells were plated on glass coverslips and grown as described above. To inhibit the karyopherin-dependent nuclear import pathway, cells were transfected and then treated with 25 μM ivermectin (IVM) for 4 h. Cells then were fixed with 4% PFA, counter-stained with DAPI, and imaged using a Zeiss LSM 880 laser scanning confocal microscope equipped with a 63×/1.4 Apochromat Oil DIC objective. The results were evaluated using the student’s *t*-test. GraphPad Prism (RRID: SCR_002798) was used for statistical analysis and generating graphs. Data were considered statistically significant when *p* < 0.05.

## Supplementary Material



## Notes

Results of the cell cycle synchronization (Supplementary Figure 1), evaluation of colocalization in cells transfected with nuclear export signal-deletion constructs of desmin (Supplementary Figure 2) and comparative sequence alignment of desmin across species highlighting the conservation of the proposed NLS regions (Supplementary Figure 3). Details and results of the fractionation experiment (PDF)
