# Supplementary material for: Identification and Functional Analysis of Dual Nuclear Localization Signals on Desmin
Source: ACS Omega. 2026 Jan 23;11(5):7257–68. doi: 10.1021/acsomega.5c07336 (PMC13105260; doi:10.1021/acsomega.5c07336)
Supplement: Supplementary file 1 [file ao5c07336_si_001.pdf]

COVER SHEET

Authors: Ecem KURAL MANGIT[a,b], Pervin DİNÇER[a] *

Manuscript Title: Identification and Functional Analysis of Dual Nuclear Localization Signals on Desmin

Affiliations and e-mail addresses:

Ecem KURAL MANGIT:

ecemkural@hacettepe.edu.tr

[a]Hacettepe University, Department of Medical Biology, Faculty of Medicine, Ankara, 06100, Turkey

[b]Hacettepe University, Laboratory Animals Research and Application Center, Ankara, 06100, Turkey

Pervin DİNÇER:
pdincer@hacettepe.edu.tr

[a]Hacettepe University, Department of Medical Biology, Faculty of Medicine, Ankara, 06100, Turkey

Corresponding Author:

Pervin DİNÇER:
Email: pdincer@hacettepe.edu.tr

Phone: +905322626883

Hacettepe University, Department of Medical Biology, Faculty of Medicine, Ankara, 06100, Turkey

Supplementary Figure 1: Cells following cell cycle synchronization. **A.** Representative images of synchronized cells at 24, 48, and 72 hours after transfer into growth medium. For comparison, control cells cultured under standard conditions are presented at the corresponding time points. Scale bar: 1 cm. B. Flow cytometry analysis. The gating strategy used for data analysis in flow cytometry is presented on the left side. On the right, the upper panel shows histograms of synchronized cells, whereas the lower panel depicts control cells. Cell cycle analysis revealed marked differences between starved and non-starved cultures. In the absence of starvation, the distribution was 15.9% in G0/G1 (2N), 14.6% in S phase, and 64.0% in G2/M (4N), indicating a predominance of cells with doubled DNA content. Following starvation, the proportion of cells in G0/G1 increased to 54.1%, while the S-phase population decreased slightly to 11.9%. Conversely, the G2/M fraction was markedly reduced to 13.0%. These shifts are consistent with a starvation-induced G1 arrest, characterized by accumulation of cells with 2N DNA content and depletion of the G2/M population.

A.

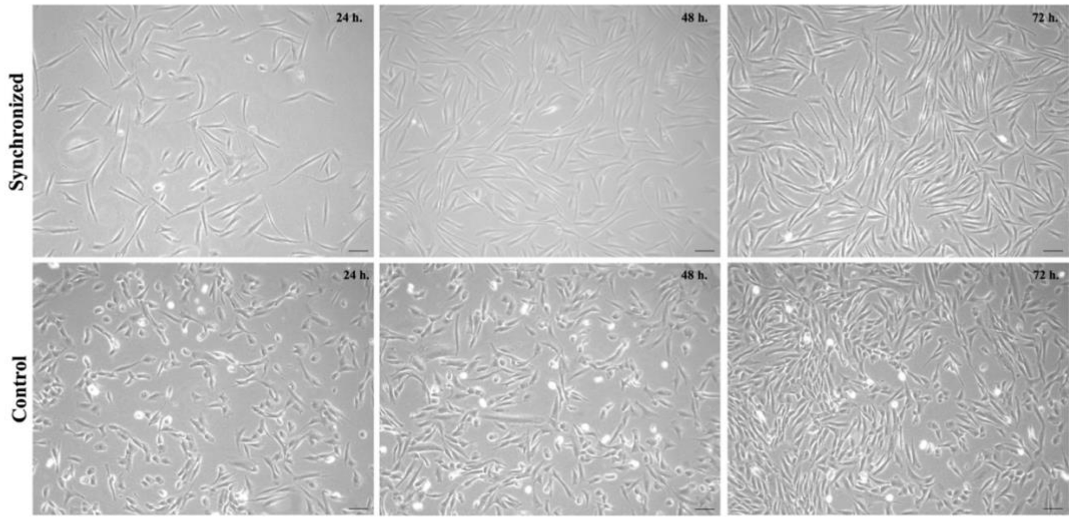

B.

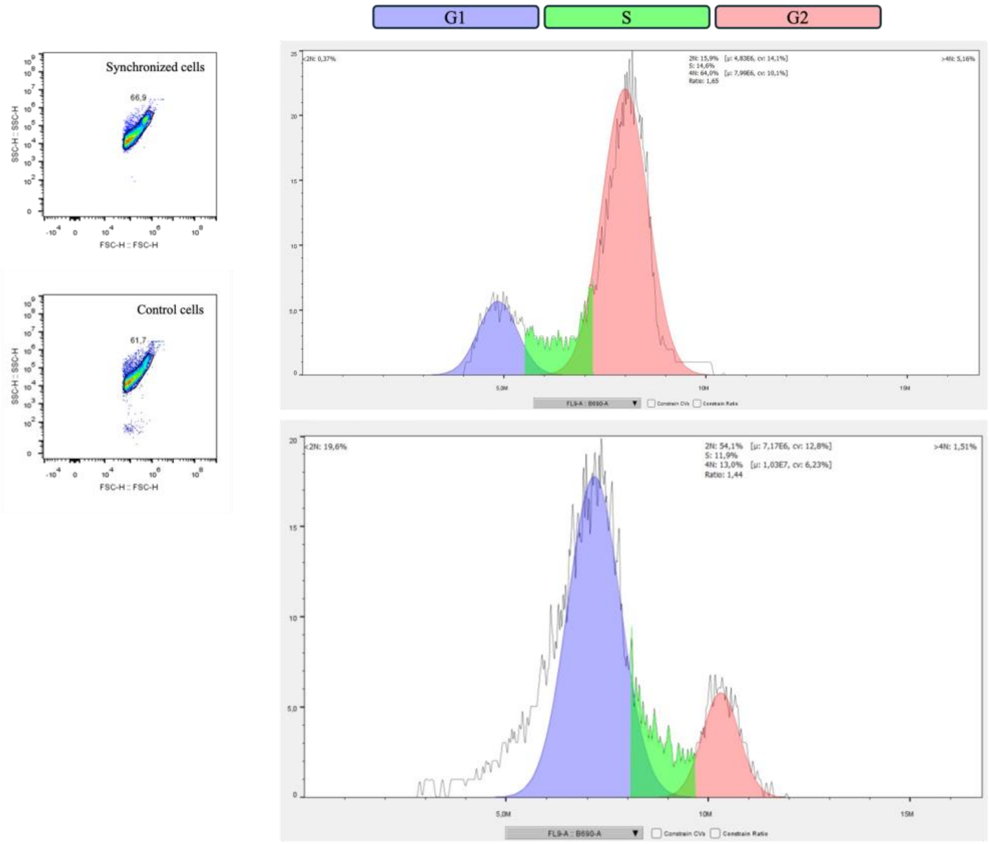

Phase	Before Starvation	After Starvation	Change
2N (G0/G1)	15.9% (μ: 4.83E6, CV: 14.1%)	54.1% (μ: 7.17E6, CV: 12.8%)	↑ increase (~3.4-fold)
S phase	14.60%	11.90%	↓ decrease (~18%)
4N (G2/M)	64.0% (μ: 7.99E6, CV: 10.1%)	13.0% (μ: 1.03E6, CV: 6.23%)	↓↓ strong decrease (~80%)
Ratio	1.65	1.44	↓ decrease

Supplementary Figure 2: Evaluation of colocalization in cells transfected with *desmin mutant lacking the signal sequence located between amino acids 192–200.* compared to full-length desmin sequence. The Mann-Whitney test was used to compare the PCC values.

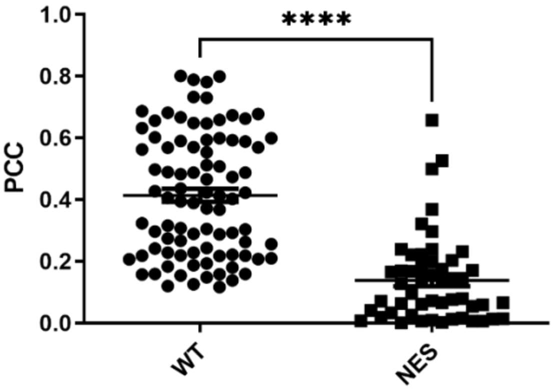

P< 0.0001

Supplementary Figure 3: Comparative sequence alignment of desmin across species highlighting the conservation of the proposed NLS regions. Desmin protein sequences from zebrafish (*Danio rerio*; Uniprot ID: F1R8W4), chicken (*Gallus gallus*; Uniprot ID: P02542), mouse (*Mus musculus*; Uniprot ID: P31001), monkey (*Macaca fascicularis*; Uniprot ID: A0A2K5TNF9), and human (*Homo sapiens*; Uniprot ID: P17661) were aligned using the UniProt Align tool (https://www.uniprot.org/align). The regions corresponding to the candidate NLSs are indicated by red boxes.

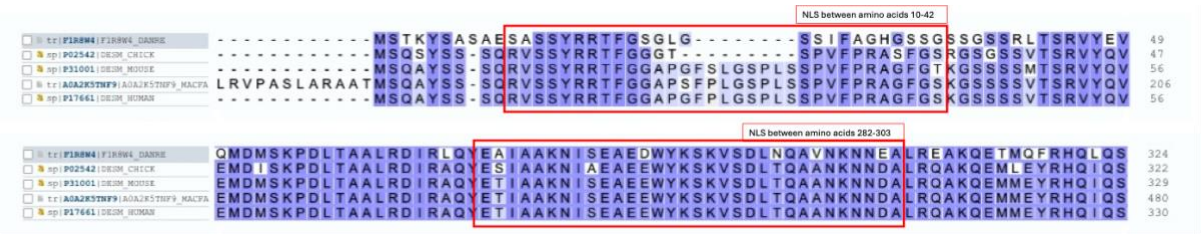

Fractionation experiments

To examine the potential nuclear localization of desmin in the nucleus, we attempted to isolate proteins separately from nuclear and cytoplasmic fractions using three different approaches. In each case, varying degrees of cytoplasmic contamination were observed in the nuclear fraction. Representative blots are provided in Supplementary Figure 4 to illustrate the methodological limitations.

In the first approach, adapted from Fuchs (2016) [1], cells were detached using trypsin, and cellular debris was removed by centrifugation. The cell pellet was resuspended in protein isolation buffer (20 mM Tris-HCl, pH 7.6; 50 mM β-mercaptoethanol; 0.1 mM EDTA; 2 mM $MgCl_2$; protease inhibitor cocktail) and incubated for 2 minutes at room temperature followed by 10 minutes at 4°C. For cytoplasmic protein isolation, 10% NP-40 was added to the protein isolation buffer, and cells were lysed by gentle pipetting. Lysates were centrifuged at 600 *g* for 5 minutes, and the cytoplasmic fraction was collected in a clean tube. For nuclear protein isolation, the nuclear-containing pellet was resuspended in RIPA buffer. Following sonication, samples were centrifuged at 17 000 *g* for 15 minutes, and the supernatant containing nuclear proteins was collected. β-actin (UniProt ID: P60709) was initially employed as a cytoplasmic marker. However, its presence in both fractions prompted a literature search, which revealed that β-actin can localize to both the cytoplasm and the nucleus [2–4]. An alternative cytoplasmic marker was therefore considered, and membranes were subsequently probed with desmin. Desmin was detected at comparable levels in both fractions, which we interpreted as reflecting limitations in the efficiency of nuclear-cytoplasmic separation (Figure S4A). This conclusion was based on the fact that desmin is a major cytoplasmic structural protein in skeletal muscle; therefore, an equal distribution across both compartments would not be expected under ideal conditions. Increasing buffer stringency by replacing NP-40 with 5% SDS did not improve the separation, again suggesting constraints in fractionation specificity.

Subsequently, a commercial kit (Thermo Scientific™, NE-PER™ Nuclear and Cytoplasmic Extraction Reagents, 78833) was used for fractionation according to the manufacturer's instructions. Briefly $2x10^6$ cells were harvested using trypsin-EDTA, followed by centrifugation at 500 × g for 5 minutes. The resulting cell pellet was washed by resuspension in PBS. Cells were transferred to a 1.5 mL microcentrifuge tube and pelleted by centrifugation at 500 × g for 2–3 minutes. The supernatant was carefully removed using a pipette, leaving the cell pellet as dry as possible. 500 ul of ice-cold CER I was then added to the cell pellet. Subsequent cytoplasmic and nuclear protein extraction was performed, maintaining the volume ratio of CER I, CER II, and NER reagents at 200:11:100 µL, respectively as instructed by the manufacturer. The cell pellet was vigorously vortexed on the highest setting for 15 seconds to ensure complete resuspension and subsequently incubated on ice for 10 minutes. Ice-cold CER II was then added, and the sample was vortexed for 5 seconds followed by a 1-minute incubation on ice. After an additional 5-second vortex, the sample was centrifuged at maximum speed 17 000 × g for 5 minutes. The resulting supernatant, representing the cytoplasmic fraction, was carefully transferred to a pre-chilled tube. The remaining insoluble pellet, containing nuclei, was resuspended in ice-cold NER and vortexed for 15 seconds. The sample was incubated on ice with intermittent vortexing for 15 seconds every 10 minutes over a total duration of 40 minutes. Following centrifugation at maximum speed 17 000 × g for 10 minutes, the nuclear extract was collected. Lamin B was employed as the nuclear marker. Strong lamin B signals in both fractions suggested incomplete resolution of nuclear and cytoplasmic proteins (Figure S4B). To minimize cytoplasmic carryover, an additional centrifugation and PBS wash were applied as suggested by the manufacturer. Despite these adjustments, desmin was consistently detected in both fractions, with a stronger signal in the nuclear fraction. Increasing vortexing and incubation times to enhance compartmental separation yielded similar results, again indicating methodological limitations.

Finally, we employed the method described by Dimauro (2012) [5] with a small adjustment; a manual Dounce homogenizer was used instead of an automatic one as in the original protocol. In this protocol, proteins were obtained through sequential buffer treatments and centrifugation steps following homogenization. As nuclear–cytoplasmic separation primarily relies on the homogenization step in this protocol, the initial disruption was carried out with varying stroke numbers, while the process was carefully monitored under a microscope to ensure to protect nuclear integrity to avoid contamination. We used $2x10^6$ cells and applied 35, 43 and 47 strokes. Desmin was again detected at comparable levels in both fractions (Figure S4C). Modifying the buffer composition by adding 2% SDS (250 mM sucrose, 50 mM Tris–HCl pH 7.4, 5 mM $MgCl_2$, protease and phosphatase inhibitor cocktails) did not alter the outcome, as desmin remained detectable in both fractions, further illustrating methodological limitations.

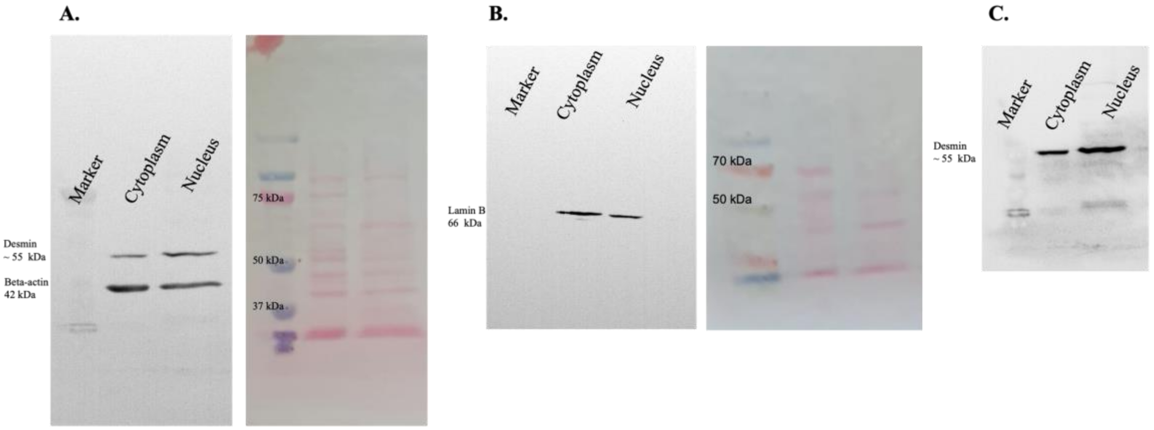

Supplementary Figure 4: Representative images of different fractionation approaches. A. Representative Western blot image of the method adapted from Fuchs (2016) [1]. Left panel: antibody-probed membrane; right panel: Ponceau staining. B. Representative Western blot image of the experiments performed using the commercial kit. Left panel: antibody-probed membrane; right panel: Ponceau staining. C. Representative Western blot image of the method adapted from Dimauro (2012) [5]. Ponceau staining was not performed for this experiment.

Taken together, across all tested approaches desmin was consistently detected in both nuclear and cytoplasmic fractions. These findings do not conclusively establish nuclear localization but rather highlight the technical constraints and methodological limitations of fractionation procedures in this cellular context. Therefore, these experiments are presented as Supplementary Data to illustrate the limitations encountered with different fractionation approaches rather than to provide definitive evidence for nuclear localization of desmin."

References

1. Fuchs C, Gawlas S, Heher P, Nikouli S, Paar H, Ivankovic M, et al. Desmin enters the nucleus of cardiac stem cells and modulates Nkx2.5 expression by participating in transcription factor complexes that interact with the nkx2.5 gene. Biol Open. 2016;5(2):140–53.

2. Zhang S, Buder K, Burkhardt C, Schlott B, Görlach M, Grosse F. Nuclear DNA Helicase II/RNA Helicase A Binds to Filamentous Actin *. J Biol Chem. 2002 Jan 4;277(1):843–53.

3. J⊘nson L, Vikesaa J, Krogh A, Nielsen LK, Hansen T vO, Borup R, et al. Molecular Composition of IMP1 Ribonucleoprotein Granules *. Mol Cell Proteomics. 2007 May 1;6(5):798–811.

4. Schrank BR, Aparicio T, Li Y, Chang W, Chait BT, Gundersen GG, et al. Nuclear Arp2/3 drives DNA break clustering for homology-directed repair. Nature. 2018 July;559(7712):61–6.

5. Dimauro I, Pearson T, Caporossi D, Jackson MJ. A simple protocol for the subcellular fractionation of skeletal muscle cells and tissue. BMC Res Notes. 2012 Sept 20;5(1):513.
